# Surveillance for Waterborne Disease Outbreaks Associated with Drinking Water — United States, 2011–2012

**DOI:** 10.15585/mmwr.mm6431a2

**Published:** 2015-08-14

**Authors:** Karlyn D. Beer, Julia W. Gargano, Virginia A. Roberts, Vincent R. Hill, Laurel E. Garrison, Preeta K. Kutty, Elizabeth D. Hilborn, Timothy J. Wade, Kathleen E. Fullerton, Jonathan S. Yoder

**Affiliations:** 1 Epidemic Intelligence Service, CDC; 2 Division of Foodborne, Waterborne, and Environmental Diseases, National Center for Emerging and Zoonotic Infectious Diseases, CDC; 3 Division of Bacterial Diseases, National Center for Immunization and Respiratory Diseases, CDC; 4 U.S. Environmental Protection Agency

Advances in water management and sanitation have substantially reduced waterborne disease in the United States, although outbreaks continue to occur (1). Public health agencies in the U.S. states and territories* report information on waterborne disease outbreaks to the CDC Waterborne Disease and Outbreak Surveillance System (http://www.cdc.gov/healthywater/surveillance/index.html). For 2011–2012, 32 drinking water–associated outbreaks were reported, accounting for at least 431 cases of illness, 102 hospitalizations, and 14 deaths. *Legionella* was responsible for 66% of outbreaks and 26% of illnesses, and viruses and non-*Legionella* bacteria together accounted for 16% of outbreaks and 53% of illnesses. The two most commonly identified deficiencies† leading to drinking water–associated outbreaks were *Legionella* in building plumbing§ systems (66%) and untreated groundwater (13%). Continued vigilance by public health, regulatory, and industry professionals to identify and correct deficiencies associated with building plumbing systems and groundwater systems could prevent most reported outbreaks and illnesses associated with drinking water systems.

This report provides information on drinking water–associated¶ waterborne disease outbreaks in which the first illness occurred in 2011 or 2012** (http://www.cdc.gov/healthywater/surveillance/drinking-surveillance-reports.html), and summarizes outbreaks reported to the Waterborne Disease and Outbreak Surveillance System through the electronic National Outbreak Reporting System (http://www.cdc.gov/nors/about.html) as of October 30, 2014. For an event to be defined as a waterborne disease outbreak, two or more persons must be linked epidemiologically by time, location of water exposure, and case illness characteristics; and the epidemiologic evidence must implicate water as the probable source of illness. Data submitted for each outbreak include 1) the number of cases, hospitalizations, and deaths; 2) the etiologic agent (confirmed or suspected); 3) the implicated water system; 4) contributing factors in the outbreak; and 5) the setting of exposure.

Public health officials from 14 states reported 32 outbreaks associated with drinking water during the time period (Table 1) (http://www.cdc.gov/healthywater/surveillance/drinking-water-tables-figures.html). These outbreaks resulted in at least 431 cases, 102 hospitalizations (24% of cases), and 14 deaths. At least one etiologic agent was identified in 30 (94%) outbreaks. *Legionella* was implicated in 21 (66%) outbreaks, 111 (26%) cases, 91 (89%) hospitalizations, and all 14 deaths. Norovirus was implicated in two single-etiology outbreaks involving 138 cases, with no hospitalizations or deaths. Three outbreaks caused by non-*Legionella* bacteria resulted in 90 (21%) cases, among which 56 (62%) were caused by Shiga toxin–producing *Escherichia coli*, 22 (24%) by *Shigella sonnei*, and 12 (13%) by *Pantoea agglomerans* (hospital-acquired bloodstream infection). Common exposure settings among drinking water–associated outbreaks were hospitals or health care facilities (n = 16, 50%), hotels (n = four, 13%), and camps/cabins (n = three, 9%). The number and etiological categories of drinking water–associated outbreaks reported every year since 1971 were reviewed for comparison (Figure).

The etiologies, water systems, water sources, predominant illness types, and deficiencies identified for drinking water–associated outbreaks and outbreak-associated cases were ranked in order of frequency (Table 2). *Legionella* was the most frequently reported outbreak etiology (65.6%), thus acute respiratory illness was the most commonly reported illness type. Outbreaks associated with community water systems†† (78.1%) outnumbered those associated with noncommunity systems and bottled water. Outbreaks associated with water systems that used surface water sources (56.3%) were more frequently reported than outbreaks associated with all other sources. The deficiency that led to most drinking water–associated outbreaks (n = 21, 65.6%) was the presence of *Legionella* in drinking water systems. The second most common deficiency was untreated groundwater (i.e., groundwater contamination at the source), both alone (n = four, 12.5%) and in combination with untreated surface water (n = one, 3.1%). All five drinking water–associated outbreaks with groundwater deficiencies (including one outbreak with multiple deficiencies) occurred in noncommunity water systems; four occurred in camps or outdoor workplaces and one occurred in a meeting facility. No reported outbreaks occurred in individual water systems (e.g., private wells).

Among 431 cases attributed to drinking water–associated outbreaks, the etiologies, illnesses, water sources and systems, and deficiencies were distributed differently than among the related outbreaks. Viruses caused 32.0% of cases, followed by *Legionella* (25.8%), and non-*Legionella* bacteria (20.9%). Over half of cases (51.5%) were linked to noncommunity water systems, and cases linked to groundwater (60.6%) were more frequently reported than all other reported sources. Most cases involved acute gastrointestinal illness (71.5%). Together, deficiencies of untreated groundwater and *Legionella* in drinking water systems accounted for 72.4% of all outbreak-associated cases.

Data were received concerning two previously unreported outbreaks with onset dates of first illness in 2009 (Table 1). These outbreaks were caused by *Legionella pneumophila* serogroup 1, and resulted in 14 cases, eight hospitalizations and one death. Data on these two outbreaks are presented (Table 1) (Figure) but are not included in the analysis of outbreaks that occurred in 2011 and 2012.

## Discussion

Since the early 20th century, water treatment processes and regulations have greatly reduced the transmission of pathogens through public drinking water supplies in the United States (1). The outbreaks reported during this surveillance period highlight several emerging and persisting public health challenges associated with drinking water systems. First, *Legionella* is the most frequently reported etiology among drinking water outbreaks; it is typically acquired through inhalation of aerosolized water containing the organism. All 14 outbreak-associated deaths reported were caused by *Legionella*, including 12 (86%) cases associated with health care facilities. Therefore, improved *Legionella* control and mitigation are needed, especially in health care settings. Second, chlorine-sensitive, gastrointestinal pathogens (norovirus, non-*Legionella* bacteria, *Giardia*§§) accounted for more than half of drinking water outbreak-associated cases, even though they only caused eight outbreaks. The comparatively high morbidity that accompanied these outbreaks highlights the importance of source water monitoring, adequate initial disinfection, and maintaining sufficient levels of disinfectant throughout a system at all times when indicated by the results of monitoring and risk analyses (2). Finally, the increase in cases that accompanied drinking water–associated outbreaks in noncommunity water systems,¶¶ from 15% in 2009–2010 to 52% in 2011–2012, indicates that additional efforts are needed to prevent outbreaks associated with these small-scale, typically intermittently used systems; full implementation of the Environmental Protection Agency (EPA) Ground Water Rule and Revised Total Coliform Rule,*** might mitigate vulnerabilities in these systems in the future (2,3).

Although the total number of drinking water–associated outbreaks has remained nearly constant (36 in 2007–2008, 35 in 2009–2010, and 32 in 2011–2012), *Legionella* has caused increasing proportions of drinking water–associated outbreaks (33%, 60%, and 66% during each of these time periods, respectively) (4,5). This pattern has been driven by the increasing proportion of *Legionella* outbreaks among those in community water systems (60%, 76%, and 84% during each of these time periods, respectively) (4,5). In 2011–2012, among 21 *Legionella* outbreaks in community water systems, 14 (67%) occurred in hospitals or health care facilities, illustrating the disproportionate disease burden among hospitalized persons, who are more likely to be older or have underlying conditions that increase their risk of developing Legionnaire’s disease (6). *Legionella* outbreaks are particularly challenging to prevent and control, in part because the organism lives and multiplies in building plumbing systems, which usually fall outside water utility and regulatory oversight (6,7). One *Legionella* outbreak occurred in a hotel that used point-of-entry water filters, which effectively dechlorinated all water entering the building, and illustrates the importance of maintaining sufficient residual disinfectant in plumbing systems.

The five drinking water–associated outbreaks and 222 outbreak-associated cases from noncommunity water systems reported for 2011–2012 represented an increase since 2009–2010, illustrating two additional public health challenges beyond *Legionella*. First, the etiologies in these outbreaks were varied but were predominantly norovirus, non-*Legionella* bacteria and *Giardia.* Moreover, the majority of cases caused by these pathogens occurred during the five outbreaks associated with noncommunity systems. Second, all five noncommunity outbreaks originated from groundwater sources. Specifically, four occurred in outdoor camp or work settings where a source spring was contaminated directly or by inflow from a stream, and the fifth occurred at a meeting facility where a well was contaminated with septic tank overflow. Because these outbreaks share common settings, water system types, and chlorine-sensitive pathogens, a large potential reduction in gastrointestinal illnesses is possible when noncommunity groundwater systems are properly maintained and operated to reduce or inactivate microbial contamination. In addition, these outbreaks underscore the importance of protecting groundwater sources from fecal contamination. Groundwater source protection will be enhanced by improved awareness of and full compliance with protective regulations, such as EPA’s Ground Water Rule and Revised Total Coliforms Rule (2,3). However, EPA lacks authority to regulate private wells or onsite wastewater systems (i.e., septic systems) not connected to public water or wastewater systems. Septic systems are used in 20% of U.S. homes, and each year 10%–20% of septic systems malfunction (8). Improper design, maintenance, or location of private wells and septic systems contributed to 67% of reported outbreaks from groundwater contamination from 1971–2008 (9), but these outbreaks can be avoided with proper design and regular service and maintenance as recommended by EPA (8).

**Summary**

What is already known on this topic?

Waterborne disease outbreaks associated with drinking water continue to occur in the United States. CDC collects data on waterborne disease outbreaks submitted from all states and territories through the Waterborne Disease and Outbreak Surveillance System.

What is added by this report?

During 2011–2012, a total of 32 drinking water–associated outbreaks were reported to CDC, resulting in 431 cases of illness, 102 hospitalizations, and 14 deaths. *Legionella* accounted for 66% of outbreaks and 26% of illnesses, and viruses and non-*Legionella* bacteria together accounted for 16% of outbreaks and 53% of illnesses. The two most commonly identified deficiencies leading to drinking water–associated outbreaks were *Legionella* in building plumbing systems (66%) and untreated groundwater (13%).

What are the implications for public health practice?

Efforts to identify and correct the deficiencies implicated in drinking water–associated outbreaks, particularly *Legionella* growth in plumbing systems, and contaminated groundwater, could prevent many outbreaks and illnesses. Additional research is needed to understand the interventions and regulations that are most effective for controlling the growth of *Legionella* and for reducing outbreaks of legionellosis.

The findings in this report are subject to at least two limitations. First, the detection and investigation of outbreaks might be incomplete, for several reasons. Linking illness to drinking water is inherently difficult through outbreak investigation methods (e.g., case-control and cohort studies) because most persons have daily exposure to tap water (10). The capacity to conduct environmental investigations that can provide information on water system deficiencies contributing to outbreaks, and strengthen evidence implicating drinking water as a common source of infection, might vary by state and locality. Second, the level of surveillance and reporting activity, as well as reporting requirements, vary across states and localities. For these reasons, outbreak surveillance data underestimate actual values, and should not be used to estimate the total number of outbreaks or cases of waterborne disease.

Compared with the previous 2-year reporting period (2009–2010), the proportion of outbreaks with deficiencies in the federally regulated portions of public water systems (i.e., up to the water meter or property line) during 2011–2012 has declined from 46% to 20%. Nonetheless, challenges with noncommunity water systems are ongoing, and efforts to prevent illnesses associated with untreated groundwater are needed. Furthermore, deficiencies at non-federally (i.e., not under jurisdiction of water utilities or EPA) regulated points, such as private wells and building plumbing systems, are also increasingly reported to cause illness, especially legionellosis. Of additional concern is the likelihood that, as older age is a risk factor for Legionnaire’s disease (6), an aging U.S. population will result in an increased proportion of individuals at higher risk. Expanded partnerships between public health, regulatory, and industry professionals to develop and use both regulatory and nonregulatory approaches to identify and address groundwater and building plumbing system deficiencies could prevent most reported outbreaks associated with drinking water systems.

## Figures and Tables

**FIGURE:**
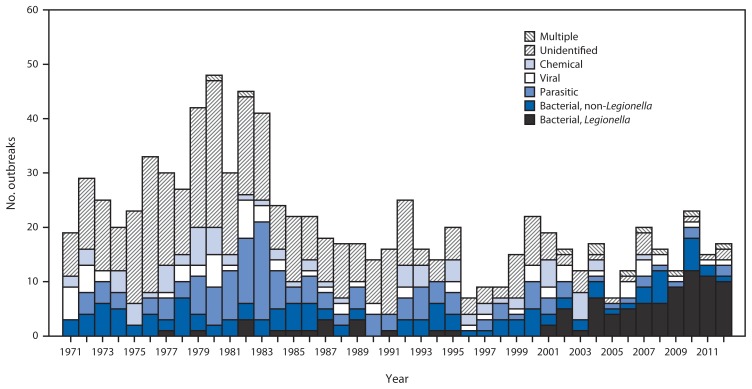
Etiology of 885 drinking water–associated outbreaks, by year — United States, 1971–2012* * Legionellosis outbreaks were first reported to CDC Waterborne Disease and Outbreak Surveillance System in 2001; Legionellosis outbreaks before 2001 were added retrospectively during the 2007–2008 reporting period.

**TABLE 1:** Waterborne disease outbreaks associated with drinking water (N = 32), by state/jurisdiction and month of first case onset — Waterborne Disease and Outbreak Surveillance System, United States, 2011–2012

State/ Jurisdiction	Month	Year	Etiology*	Predominant illness†	No. cases	No. hospitalizations§	No. deaths¶	Water system**	Water source	Setting
Alaska	Jun	2012	*Giardia intestinalis*	AGI	21	0	0	Transient noncommunity	Spring, Well, River/Stream††	Camp/Cabin
Arizona	Mar	2011	Unknown	AGI	3	0	0	Nontransient noncommunity	Spring	Outdoor workplace
Colorado	Oct	2012	Propylene glycol suspected§§	AGI	7	0	0	Community	Lake/Reservoir/Impoundment	Hospital/Health care
Florida	Aug	2009¶¶	*L. pneumophila* serogroup 1	ARI	10	4	1	Community	Unknown	Hotel/Motel/Lodge/Inn
Florida	Jul	2011	*Shigella sonnei* subgroup D	AGI	22	0	0	Commercially bottled	Unknown	Indoor workplace/Office
Florida	Mar	2012	Unknown***	AGI	3	0	0	Commercially bottled	Well	Indoor workplace/Office
Idaho	May	2012	*Campylobacter, Giardia intestinalis*	AGI	7	0	0	Community	River/Stream/Well	Community/Municipality
Illinois	Aug	2012	*Pantoea agglomerans* †††	Other	12	9	0	Community	Lake/Reservoir/Impoundment	Hospital/Health care
Maryland	May	2011	*L. pneumophila* serogroup 1	ARI	7	6	1	Community	Well	Hotel/Motel/Lodge/Inn
Maryland	May	2012	*L. pneumophila* serogroup 1	ARI	3	2	1	Community	Lake/Reservoir/Impoundment	Hospital/Health care
New Mexico	Jun	2011	Norovirus	AGI	119	0	0	Transient noncommunity	Spring§§§	Camp/Cabin
New York	Apr	2009¶¶¶	*L. pneumophila* serogroup 1	ARI	4	4	0	Community	Lake/Reservoir/Impoundment	Apartment/Condo
New York	Jun	2011	*L. pneumophila serogroup 1*	ARI	2	2		Community	River/Stream	Hospital/Health care
New York	Sep	2011	*L. pneumophila* serogroup 1	ARI	12	10	0	Community	Lake/Reservoir/Impoundment	Hotel/Motel/Lodge/Inn
New York	Sep	2011	*L. pneumophila* serogroup 1	ARI	3		0	Community	Lake/Reservoir/Impoundment	Hospital/Health care
New York	Jan	2012	*L. pneumophila* serogroup 1	ARI	3			Community	Lake/Reservoir/Impoundment	Hotel/Motel/Lodge/Inn
New York	Mar	2012	*L. pneumophila* serogroup 1	ARI	2	1	0	Community	Lake/Reservoir/Impoundment	Hospital/Health care
New York	Apr	2012	*L. pneumophila* serogroup 1	ARI	2	2		Community	Lake/Reservoir/Impoundment	Apartment/Condo
New York	Oct	2012	*L. pneumophila* serogroup 1	ARI	2	1	0	Community	Lake/Reservoir/Impoundment	Hospital/Health care
New York	Nov	2012	*L. pneumophila* serogroup 1	ARI	2	2	0	Community	Lake/Reservoir/Impoundment	Hospital/Health care
Ohio	Jan	2011	*L. pneumophila* serogroup 1	ARI	11	11	1	Community	Well	Hospital/Health care
Ohio	Mar	2011	*L. pneumophila* serogroup 1	ARI	8	7	0	Community	Lake/reservoir/impoundment	Hospital/Health care
Ohio	Aug	2011	*L. pneumophila*	ARI	10	4	2	Community	Lake/Reservoir/Impoundment	Hospital/Health care
Ohio	Nov	2012	*L. pneumophila* serogroup 1	ARI	2	2	0	Community	Lake/Reservoir/Impoundment	Hospital/Health care
Pennsylvania	Feb	2011	*L. pneumophila* serogroup 1	ARI	22	22	5	Community	Lake/Reservoir/Impoundment	Hospital/Health care****
Pennsylvania	May	2011	*L. pneumophila* serogroup 1	ARI	2	2	0	Community	Well	Long-term care facility
Pennsylvania	Aug	2011	*L. pneumophila* serogroup 1	ARI	6	5	1	Community	Well	Hospital/Health care
Pennsylvania	Mar	2012	*L. pneumophila*	ARI	2	2	1	Community	Lake/Reservoir/Impoundment	Hospital/Health care
Pennsylvania	Nov	2012	*L. pneumophila* serogroup 1	ARI	4	4	1	Community	River/Stream	Apartment/Condo
Utah	Aug	2011	STEC O121, STEC O157:H7	AGI††††	56	2	0	Transient noncommunity	Spring	Camp/Cabin
Utah	Jul	2012	*L. pneumophila* serogroup 1	ARI	3	3	0	Community	Lake/Reservoir/Impoundment	Hotel/Motel/Lodge/Inn
Utah	Aug	2012	*Giardia intestinalis*	AGI	28	0	0	Community	Well	Subdivision/Neighborhood
Washington	Jan	2011	*L. pneumophila* serogroup 1	ARI	3	3	1	Community	Well	Hospital/Health care
Wisconsin	Aug	2012	Norovirus Genogroup I.2	AGI	19	0	0	Transient noncommunity	Well§§§§	Hall/Meeting facility

**Abbreviations:** AGI = acute gastrointestinal illness; ARI = acute respiratory illness; *L. pneumophila* = *Legionella pneumophila*; other = undefined, illnesses, conditions, or symptoms that cannot be categorized as gastrointestinal, respiratory, ear-related, eye-related, skin-related, neurologic, hepatitis, or caused by leptospirosis; STEC = Shiga toxin–producing *Escherichia coli*.

* Etiologies listed are confirmed, unless indicated “suspected.” For multiple-etiology outbreaks, etiologies are listed in alphabetical order.

† The category of illness reported by =50% of ill respondents. All legionellosis outbreaks were categorized as ARI.

§ Value was set to “missing” in reports where zero hospitalizations were reported and the number of people for whom information was available was also zero.

¶ Value was set to “missing” in reports where zero deaths were reported and the number of people for whom information was available was also zero.

** Community and noncommunity water systems are public water systems that have =15 service connections or serve an average of =25 residents for =60 days/year. A community water system serves year-round residents of a community, subdivision, or mobile home park. A noncommunity water system serves an institution, industry, camp, park, hotel, or business and can be nontransient or transient. Nontransient systems serve =25 of the same persons for =6 months of the year but not year-round (e.g., factories and schools) whereas transient systems provide water to places in which persons do not remain for long periods of time (e.g., restaurants, highway rest stations, and parks). Water systems in this table include community, noncommunity and bottled.

†† Spring water source contaminated during temporary connection with contaminated surface water source (stream).

§§ Skin and eye symptoms in addition to AGI; other possible chemical exposures from cross contamination between drinking water and boiler water.

¶¶ The first case of illness in this outbreak occurred before 2011–2012, but the outbreak was reported later and not previously described in a surveillance report.

*** Chemical contamination suspected due to short incubation period; three bottled water samples tested, no chemical contamination detected.

††† Outbreak of Pantoea agglomerans bloodstream infection in a health care facility linked to the drinking water system. Oncology clinic patients received infusions contaminated with P. agglomerans via central line, and environmental samples from the clinic and pharmacy where infusions were prepared shared the PFGE pattern found in patient blood samples. P. agglomerans was isolated from the pharmacy sink where the infusates were prepared, as well as from the oncology clinic icemaker. This is the first report of a Pantoea infection outbreak in a health care facility, and in a drinking water-associated outbreak surveillance report.

§§§ Outbreak occurred at the same venue with same etiology and water source as an outbreak previously reported in 1999; contamination by surface water was suspected, based on the 1999 investigation.

¶¶¶ The first ill cases were identified in 2009, and were linked by molecular subtyping in 2012 to additional ill individuals living in the same apartment complex with onset dates in 2011 and 2012.

**** Hospital had a copper/silver ionization system, with concentrations at manufacturer-recommended levels, in place to control Legionella at the time of the outbreak.

†††† No outbreak-associated cases of hemolytic uremic syndrome (HUS) were reported.

§§§§ Setting was a meeting facility, where owner was unaware of and not maintaining septic system; system overflowed and contaminated the well.

**TABLE 2:** Rank order (most to least common) of etiology, water system, water source, predominant illness, and deficiencies associated with 32 drinking water outbreaks and 431 outbreak-related cases — United States, 2011–2012

		Outbreaks (N = 32)			Cases (N = 431)		
			
Characteristic	Rank	Category	No.	(%)	Category	No.	(%)
**Etiology**							
	1	Bacteria, *Legionella*	21	(65.6)	Viruses	138	(32.0)
	2	Bacteria, non-*Legionella*	3	(9.4)	Bacteria, *Legionella*	111	(25.8)
	3	Parasites	2	(6.3)	Bacteria, non-*Legionella*	90	(20.9)
	4	Viruses	2	(6.3)	Parasites	49	(11.4)
	5	Unknown	2	(6.3)	Chemical*	26	(6.0)
	6	Chemical*	1	(3.1)	Unknown	10	(2.3)
	7	Multiple†	1	(3.1)	Multiple†	7	(1.6)
**Water system** §							
	1	Community	25	(78.1)	Noncommunity	222	(51.5)
	2	Noncommunity	5	(15.6)	Community	184	(42.7)
	3	Bottled	2	(6.3)	Bottled	25	(5.8)
**Water source**							
	1	Surface water	18	(56.3)	Ground water	261	(60.6)
	2	Ground water	11	(34.4)	Surface water	120	(27.8)
	3	Mixed¶	2	(6.3)	Unknown	22	(5.1)
	4	Unknown	1	(3.1)	Mixed¶	28	(6.5)
**Predominant Illness** **							
	1	ARI	21	(65.6)	AGI	308	(71.5)
	2	AGI	10	(31.3)	ARI	111	(25.8)
	3	Other††	1	(3.1)	Other††	12	(2.8)
**Deficiency** §§							
	1	*Legionella spp*. in drinking water system¶¶	21	(65.6)	Untreated ground water***	201	(46.6)
	2	Untreated ground water***	4	(12.5)	*Legionella spp*. in drinking water system¶¶	111	(25.8)
	3	Premise plumbing system†††	2	(6.3)	Premise plumbing system	33	(7.7)
	4	Unknown/Insufficient information	2	(6.3)	Distribution system§§§	28	(6.5)
	5	Distribution system§§§	1	(3.1)	Point of use, bottled¶¶¶	22	(5.1)
	6	Multiple****	1	(3.1)	Multiple****	21	(4.9)
	7	Point of use, bottled¶¶¶	1	(3.1)	Unknown/Insufficient information	15	(3.5)

**Abbreviations:** AGI = acute gastrointestinal illness; ARI = acute respiratory illness.

* Propylene glycol detected in drinking water after cross-connection with HVAC water system.

† One outbreak had multiple etiologic agent types: Campylobacter spp. (i.e., non-Legionella bacterium) and Giardia intestinalis (i.e., parasite).

§ Community and noncommunity water systems are public water systems that have =15 service connections or serve an average of =25 residents for =60 days a year. Community water systems serve year-round residents of a community, subdivision, or mobile home park. Noncommunity water systems serve an institution, industry, camp, park, hotel, or business.

¶ Includes outbreaks with mixed water sources (i.e., ground water and surface water). Two giardiasis outbreaks were associated with mixed source community water systems.

** The category of illness reported by =50% of ill respondents; all legionellosis outbreaks were categorized as ARI.

§§ Outbreaks are assigned one or more deficiency classifications. (Source: Brunkard, JM, Ailes E, Roberts VA, et al. Surveillance for waterborne disease outbreaks associated with drinking water-United States, 2007–2008. MMWR Surveill Summ 2011;60:38–68).

†† Symptoms for one outbreak caused by Pantoea agglomerans bloodstream infection were categorized as “other.”

¶¶ Deficiency 5A. Drinking water, contamination of water at points not under the jurisdiction of a water utility or at the point of use: Legionella spp. in water system, drinking water.

*** Deficiency 2. Drinking water, contamination of water at/in the water source, treatment facility, or distribution system: untreated ground water.

††† Deficiency 6. Drinking water, contamination of water at points not under the jurisdiction of a water utility or at the point of use: Plumbing system deficiency after the water meter or property line (e.g., cross-connection, backflow, or corrosion products).

§§§ Deficiency 4. Drinking water, contamination of water at/in the water source, treatment facility, or distribution system: Distribution system deficiency, including storage (e.g., cross-connection, backflow, contamination of water mains during construction or repair).

¶¶¶ Deficiency 11C. Drinking water, contamination of water at points not under the jurisdiction of a water utility or at the point of use: Contamination at point of use, commercially bottled water.

**** Multiple deficiencies were assigned to one giardiasis outbreak which contributed 21 cases: deficiency 1, untreated surface water; and deficiency 2, untreated ground water.

## Footnotes

* Territories include the District of Columbia, Guam, Puerto Rico, the Marshall Islands, the Federated States of Micronesia, the Commonwealth of the Northern Mariana Islands, Palau, and the U.S. Virgin Islands.

† Outbreaks are assigned one or more deficiency classifications based on available data. (http://www.cdc.gov/healthywater/surveillance/deficiency-classification.html).

§ “Plumbing” refers to the pipes that are within a building or within a service line leading into a building, distinguished from the distribution system of pipes that compose the water supply.

¶ Drinking water, also called potable water, is water for human consumption (e.g., drinking, bathing, showering, hand washing, teeth brushing, food preparation, dishwashing, maintaining oral hygiene) and includes water collected, treated, stored or distributed in public and individual water systems, as well as bottled water.

** This report also includes two previously unreported outbreaks with first case onset dates in 2009.

†† Community and noncommunity water systems are public water systems that have ≥15 service connections or serve an average of ≥25 residents for ≥60 days/year. A community water system serves year-round residents of a community, subdivision, or mobile home park. A noncommunity water system serves an institution, industry, camp, park, hotel, or business and can be nontransient or transient. Nontransient systems serve ≥25 of the same persons for ≥6 months of the year but not year-round (e.g., factories and schools) whereas transient systems provide water to places in which persons do not remain for long periods of time (e.g., restaurants, highway rest stations, and parks). Individual water systems are small systems not owned or operated by a water utility that have <15 connections or serve <25 persons.

§§ One outbreak with a parasite etiology that caused 21 cases was attributed to *Giardia intestinalis*. The multiple-etiology outbreak included *Giardia* and *Campylobacter*, both of which are chlorine-sensitive.

¶¶ Groundwater (e.g., wells and springs) was the source of all outbreaks in noncommunity systems in 2011–2012.

*** EPA regulations are implemented in phases. Outbreaks reported here occurred after initial implementation (2009) but before full implementation (2014) of the Ground Water Rule, and before Revised Total Coliform Rule implementation begins (2016).
